# Supplementary material for: Enhancing the Viability of a Small Giant Panda Population Through Individual Introduction From a Larger Conspecific Group: A Scientific Simulation Study
Source: Animals (Basel). 2024 Aug 14;14(16):2345. doi: 10.3390/ani14162345 (PMC11350795; doi:10.3390/ani14162345)
Supplement: Supplementary file 1 [file animals-14-02345-s001.zip › animals-3096044-supplementary.pdf]

Table S1. Mortality of giant pandas at different ages.

Age	Female	SD	Male	SD
0~1	40.00	10.0	41.00	10.0
1~2	9.67	3.0	9.67	3.0
2~3	3.14	2.0	3.14	2.0
3~4	1.52	1.0	1.52	1.0
4~5	1.55	1.0	1.55	1.0
5~6	1.57	1.0	1.57	1.0
6~7	1.60	1.0	1.60	1.0
7~8	-	-	3.45	2.0
Adult	13.33	3.0	14.16	3.0

Table S2. Viability of small populations after individual introduction.

Introduction plan	Sex	DXL			LZP		
		P%	Gd	N	P%	Gd	N
Ideal state	—	52.5	0.6211	40	19.6	0.7443	60
Introduce 1 /2 years	F	0	0.9224	162	0	0.9349	180
	M	0	0.9148	30	0	0.9237	53
Introduce 1 /5 years	F	0.1	0.8416	75	0	0.8824	106
	M	0.5	0.7909	25	0.3	0.846	53
Introduce 2 /5 years	2F	0	0.9089	142	0	0.9247	161
	1F1M	0	0.9148	85	0	0.9237	109
	2M	0	0.8876	29	0	0.9044	54
Introduce 1 /10 years	F	7.9	0.7506	52	2.3	0.8329	79
	M	16.6	0.6979	27	6.1	0.7863	51
Introduce 2 /10 years	2F	1.4	0.8405	82	0.3	0.8837	108
	1F1M	0.5	0.8424	52	0	0.8752	81
	2M	5.2	0.7624	24	2.6	0.8342	50
Introduce 3 /10 years	3F	0.1	0.8858	113	0	0.9094	136
	2F1M	0	0.89	84	0	0.9081	108
	1F2M	0	0.8787	54	0.3	0.8978	79
	3M	1.7	0.8144	27	0.8	0.8658	51
Introduce 4 /10 years	4F	0.2	0.9072	142	—	—	—
	3F1M	0	0.9134	119	—	—	—
	2F2M	0	0.9124	86	—	—	—
	1F3M	0	0.8984	57	—	—	—
	4M	0.4	0.8503	28	—	—	—

Table S3. Viability of large populations after individual introduction.

	Introduction plan	Sex	TJH			WL		
			P%	Gd	N	P%	Gd	N
	Ideal state	—	2.4	0.8689	113	4	0.8343	90
Both apply	Introduce 1 /2 years	F	46.7	0.7124	28	66.4	0.653	17
		2F	34.1	0.7365	34	51.1	0.712	29
	Introduce 2 /5 years	1F1M	11.2	0.8138	70	20.1	0.764	55
Applicable to LZP	Introduce 3 /10 years	3F	21.2	0.7762	47	34.6	0.722	39
		2F1M	8.8	0.8261	71	12.7	0.785	56
Applicable to DXL	Introduce 4 /10 years	4F	34.6	0.7283	33	50.8	0.677	27
		3F1M	24	0.7707	48	34.7	0.731	39
		2F2M	13	0.8032	66	19.6	0.76	54
